# Coronary periarteritis and pericarditis are rare but distinct manifestations of heart involvement in IgG4-related disease: a retrospective cohort study

**DOI:** 10.1186/s13023-024-03266-y

**Published:** 2024-07-15

**Authors:** Tianrui Hua, Juan Du, Xiaoxiao Guo, Linyi Peng, Jiaxin Zhou, Yuxue Nie, Dafu Man, Mengtao Li, Lili Pan, Wen Zhang

**Affiliations:** 1 Department of Internal Medicine, Peking Union Medical College Hospital, Chinese Academy of Medical Science & Peking Union Medical College, Beijing, China; 2 Department of Rheumatology, Peking Union Medical College Hospital National Clinical Research Center for Dermatologic and Immunologic Diseases Key Laboratory of Rheumatology and Clinical Immunology, Ministry of Education, Beijing, China; 3 Department of Cardiology, Peking Union Medical College Hospital, Chinese Academy of Medical Science & Peking Union Medical College, Beijing, China; 4 The Affiliated Hospital of Inner Mongolia Medical University, Hohhot, Inner Mongolia, China; 5 Beijing AnZhen Hospital, Capital Medical University, Beijing Institute of Heart, Lung and Blood Vessel Diseases, Beijing, China

**Keywords:** IgG4-related disease, Coronary artery, Periarteritis, Pericarditis

## Abstract

**Background:**

The heart can be involved in immunoglobulin (Ig)-G4-related disease (IgG4-RD). This study aimed to summarize the clinical features and efficacy of treatment for IgG4-RD patients with heart involvement.

**Methods:**

We conducted a retrospective study enrolling 42 IgG4-RD patients with heart involvement from the IgG4-RD cohorts of the Peking Union Medical College Hospital and Beijing An Zhen Hospital, from 2010 to 2022. Clinical, laboratory, radiological data were collected, and treatment responses to glucocorticoids and immunosuppressants were analyzed.

**Results:**

IgG4-related cardiac involvement is a rare part of the IgG4-RD spectrum. The incidences of coronary periarteritis and pericarditis were 1.2%(13/1075) and 3.1%(33/1075), respectively in our cohort. Valvular disease possibly related to IgG4-RD was detected in two patients. None of the patients with myocardial involvement were identified. The average age was 58.2 ± 12.8 years, with a male predominance (76.7%). Coronary artery CT revealed that mass-like and diffuse wall-thickening lesions were the most frequently observed type of coronary periarteritis. Pericarditis presented as pericardial effusion, localized thickening, calcification and mass. After treatment with glucocorticoid and immunosuppressants, all patients achieved a reduced IgG4-RD responder index score and achieved radiological remission. Two patients with coronary peri-arteritis experienced clinical relapses during the maintenance period.

**Conclusions:**

Cardiac involvement in IgG4-RD is rare and easily overlooked since many patients are asymptomatic, and the diagnosis relies on imaging. Patients showed a satisfactory response to glucocorticoid based treatment.

**Supplementary Information:**

The online version contains supplementary material available at 10.1186/s13023-024-03266-y.

## Introduction

Immunoglobulin G4-related disease (IgG4-RD) is a systemic fibro-inflammatory disease [1–3], characterized by elevated serum IgG4 levels and massive lymphoplasmacytic infiltration in lesions containing IgG4-positive plasma cells [4]. Patients with IgG4-RD can have localized lesions or diffuse systemic disease. Previous studies have described the involvement of various organs in IgG4-RD, including autoimmune pancreatitis, sclerosing cholangitis, sialadenitis, dacryoadenitis, interstitial nephritis, lymphadenopathy, retroperitoneal fibrosis, IgG4-related hypophysitis, and IgG4-related lung disease. According to the literature, there are various types of cardiovascular involvement in IgG4-RD, including aortitis, periaortitis, periarteritis [5, 6], pericardial [7, 8] cardiac valves [9] and myocardial involvement [10]. Cardiac involvement in IgG4-RD presents with various clinical manifestations, including chest pain, dyspnea, palpitations, and heart failure. Imaging studies, including echocardiography, computed tomography, and magnetic resonance imaging, may reveal coronary artery involvement, pericardial effusion, thickening of the pericardium, myocardial nodules and thickening of cardiac valves. Histopathological examination of cardiac tissue shows infiltration of IgG4-positive plasma cells and tissue fibrosis. Coronary periarteritis is a rare manifestation of IgG4-RD that may be difficult to diagnose unless specifically sought [11], however severe consequences including myocardial infarction and heart failure could result from coronary involvement of IgG4-RD. Inflammatory pericardial fibrosis related to IgG4-RD has been demonstrated in several reports and whether IgG4-RD underlies myocardial fibrosclerosis needs further assessment. Moreover, early diagnosis and treatment are essential for minimizing irreversible organ damage or unnecessary surgical intervention [12, 13]. IgG4-RD is generally responsive to steroid therapy; however, some patients are refractory to immunosuppressive treatments or relapse after glucosteroid tapering. In this study, we aimed to explore the clinical and radiological features of patients with IgG4-RD associated cardiac manifestations and treatment strategies.

## Methods

### Patient enrollment

We performed a retrospective chart review of patients with heart involvement at Peking Union Medical College Hospital and Anzhen Hospital in Beijing, China, between 2010 and 2022. Participants fulfilled either the 2019 American College of Rheumatology (ACR)/European League Against Rheumatism (EULAR) classification criteria for IgG4-RD [14] or the 2020 revised comprehensive diagnostic criteria for IgG4-RD (definite, probable, and possible) [15, 16]. All patients signed informed consent forms. The present study (no. S-442) was approved by the Ethics Committee of the Peking Union Medical College Hospital and registered on ClinicalTrails.gov NCT01670695.

For cardiac involvement of IgG4-RD, patients needed to fulfill at least (1) or (2), plus (3): (1) histopathology: cardiac biopsy presenting with classical IgG4-RD pathology; (2) imaging: one of the following lesions revealed by echocardiography, coronary computed tomography angiography (CTA) or positron emission tomography/computed tomography (PET/CT): (a) diffuse wall thickening of coronary artery, tumorous lesions surrounding coronary artery with or without aneurysm and stenosis; (b) pericardial effusion, thickening, mass, or restrictive pericarditis; (c) cardiac valve abnormality; (d) myocardial or intracardiac mass-like lesion; [3] treatment response: the above lesions or cardiac clinical manifestations show improvement after IgG4-RD treatment.

We excluded patients with incomplete clinical data and suspected other autoimmune disorders [e.g., Sjogren’s syndrome, antineutrophil cytoplasmic antibody- (ANCA-)associated vasculitis, and sarcoidosis], suspected malignancy (lymphoma, kidney cancer, and metastatic carcinoma), and infections. In addition, coronary lesions caused only by coronary atherosclerosis were excluded.

### Clinical data, laboratory tests and imaging

Demographic data, disease duration, and accompanying involved organs were collected. Laboratory tests parameters include routine blood examination; liver and kidney functions; erythrocyte sedimentation rate (ESR); and high-sensitivity C reactive protein (hsCRP), complement, serum immunoglobulin (Ig), serum immunoglobulin G (IgG) subclass, and total IgE levels.

All patients underwent imaging examinations, such as ultrasound, computed tomography (CT) scan, and magnetic resonance imaging (MRI). Some patients underwent PET/CT. Patients with relevant symptoms underwent coronary arteriography (CAG).

Pericardial thickening is characterized by discontinuous pericardial thickening on CT. An echocardiogram simultaneously confirmed the absence of pericardial effusion to differentiate it from pericardial effusion.

### Assessment of treatment response

The overall treatment response was assessed by the changes in IgG4-RD responder index (RI) scores [17]. Clinical relapse was defined as the recurrent or new onset of clinical symptoms or imaging findings with or without elevated serum IgG4 levels. Patients were followed up at months 1, 3, and 6 and every 6 to 12 months thereafter.

### Statistical analysis

Statistical analysis was performed using SPSS version 21.0 (IBM Inc., Chicago, IL). The data are reported as the mean ± standard deviation or the median and range (interquartile range). The Student’s t-test was used for the analysis of continuous, normally distributed data, while the Mann-Whitney U test was used for the analysis of continuous nonnormally distributed data. One-way ANOVAs were used to analyze numerical variables for normally distributed data, and the Kruskal-Wallis test was used to analyze nonnormally distributed data. A P value less than 0.05 was considered statistically significant.

## Results

### Demographic and clinical features

Among all 1106 IgG4-RD patients, 1075 met overall IgG4-RD diagnosis with either the 2019 ACR/ EULAR classification criteria for IgG4-RD or the 2020 revised comprehensive diagnostic criteria for IgG4-RD, 31 were excluded for being diagnosed as IgG4-RD mimic or suspicion of other autoimmune disorders, malignancy and infection), and 5 were excluded for missing data.

A total of 42 IgG4-RD patients with heart involvement were enrolled. IgG4-related coronary periarteritis and pericarditis are rare parts of the IgG4-RD spectrum, with a prevalence of 1.2% and 3.1%, respectively, in our cohort of 1075 IgG4-RD patient. The mean (SD) age was 58.2[12.8] years and 33 (76.7%) were male. A total of 43.9% (18 of 42) of patients had an allergy history.

Of the 42 patients, 30 (71.4%) had a 2019 ACR/EULAR IgG4-RD classification score greater than 20. According to the 2020 comprehensive diagnostic criteria for IgG4-RD, 18 (42.9%) patients were diagnosed as definite IgG4-RD, 14 (33.3%) were probable, and 10 (23.8%) patients with possible IgG4-RD. Twenty-five patients underwent biopsy, including the submandibular gland, lacrimal gland, kidney, lymph node, cholecyst, mediastinum, pleura, liver, lung and hypophysis. No patient underwent coronary artery or pericardium biopsy for diagnosis.

Most patients had multiple organ involvement, except for 2 who had cardiac lesions alone, and the other 40 (95.2%) patients had at least two organs involved, including superficial and visceral organs. A total of 78.6% of patients with heart involvement had other visceral organs affected. Patients with coronary artery involvement had fewer accompanying involved organs (1, 1-3.5) than did those with pericardial involvement [2–5] (*P* = 0.064). The submandibular gland was the most commonly suffered organ (23, 54.7%), followed by the lymph nodes, and lacrimal gland (18, 42.9% and 14, 33.3%, respectively). 30.8% coronary arteritis with or without pericarditis was accompanied with abdominal aortitis. The demographic features and organ involvement of IgG4-RD with/without cardiac involvement are shown in Supplementary Table 1.

### Characteristics of IgG4-RD associated Cardiac involvement

#### Types of cardiac involvement

9 patients were diagnosed with coronary periarteritis, 29 patients had pericarditis, and 4 with both coronary periarteritis and pericarditis. In addition, 2 patients were enrolled with cardiac valves disease possibly associated with IgG4-RD. And no myocardial and intracardiac mass lesion was addressed in our patients. Clinical features were summarized in Table 1.

**Table 1:** Baseline features of IgG4-RD patients with cardiac involvements

	Total	Coronary artery	Pericardium	Both	*P*
	(*n* = 42)	(*n* = 9)	(*n* = 29)	(*n* = 4)	
Male: Female	33:9	8:1	21:8	4:00	0.323
Age of diagnosis, years	58.2 ± 12.8	60.4 ± 5.9	57.2 ± 15.0	54.3 ± 4.9	0.421
Number of involved organs	3(2-4.5)	1(1-3.5)	4 (2–5)	1.5 (0.25–5.75)	0.064
Allergy history	18/42(43.9%)	2/9(22.2%)	15/29 (51.7%)	2/4 (50.0%)	0.302
Other organs affected					
Pancreas	11/42(26.1%)	2/9(22.2%)	8/29 (27.6%)	1/4 (25.0%)	0.868
Submandibular glands	23/42(54.7%)	3/9(33.3%)	19/29 (65.5%)	2/4 (50%)	0.232
lacrimal glands	14/42(33.3%)	2/9(22.2%)	11/29 (37.9%)	2/4 (50%)	0.521
Cholangitis	6/42(14.3%)	2/9(22.2%)	3/29 (10.3%)	0/4 (0)	0.32
Sinus	6/42(14.3%)	0/9(0)	5/29 (17.2%)	1/4 (25.0%)	0.319
Abdominal periaortitis	8/42(19.0%)	2/9(22.2%)	0/29 (0)	2/4(50%)	0.046
Lung	11/42(26.2%)	0/9(0)	10/29 (34.5%)	1/4(25.0%)	0.156
Lymph nodes	18/42(42.9%)	0/9(0)	17/29 (62.1%)	1/4 (25.0%)	0.007
ACR/EULAR criteria >20	30/42(71.4%)	4/9(44.4%)	24/29 (82.8%)	2/4 (50.0%)	0.055

#### Symptoms

Among all the patients included, 31.0% (13/42) had cardiac symptoms during the course of illness. Patients with coronary arteritis presented with more cardiac symptoms (9/13, 69.2%) than those with pericardial involvement only (4/29, 13.8%). Angina was the most prevalent symptom observed; among all patients, 8 (19.0%) had angina, 4 (9.5%) presented with discomfort of the precordial area, 2 (4.8%) had palpitations, 2 (4.8%) had lower extremity edema, 2 (4.8%) had shortness of breath, and 1 (2.4%) had back pain.

#### Laboratory features

The laboratory features of cardiac involved IgG4-RD patients are summarized in Table 2. All patients with coronary artery involvement had elevated serum IgG4, 76.9% presented with hyperglobulinemia, and 76.9% had hyper-IgE. The median serum IgG4 concentration was 8490 mg/L (3630 mg/L -26,800 mg/L). The ESR and hsCRP level in patients with coronary artery involvement were 40 (20.0–66.0) mm/h and 4.9 (1.4–6.6) mg/L respectively. No significant difference was found compared with that in patients with pericardial involvement (25.0 mm/h, 10.0–71.0, and 1.5 mg/L, 0.6–11.2, respectively).

**Table 2:** Laboratory parameters of IgG4-RD with cardiac involvement

	Coronary artery (*N* = 9)	Pericardium (*N* = 29)	Both (*N* = 4)	*P* value
Hgb (g/L)	135.0(121.0-145.4)	126.0(110.5-144.5)	145.0(121.5–161.0)	0.38
WBC (10^9/L)	7.2(5.8–8.5)	6.1(4.9–9.3)	7.2(6.2–8.3)	0.77
PLT (10^9/L)	242.0(168.0-307.0)	238.0(198.5-279.5)	230.5(192.3-337.8)	0.89
EOS (%)	4.3(2.0-5.7)	3.6(1.0-7.9)	6.2(3.0-10.4)	0.48
ESR (mm/h)	40.0(20.0–66.0)	25(10.0–71.0)	20.0(3.5–62.8)	0.61
hsCRP (mg/L)	4.9(1.4–6.6)	1.5(0.6–11.2)	1.8(1.0-24.4)	0.71
IgG (g/L)	22.6(16.9–30.3)	20.9(15.0-29.8)	19.9(16.5–24.7)	0.90
IgA (g/L)	1.5(0.7–2.9)	1.5(1.2–2.2)	1.7(1.3–2.9)	0.79
IgM (g/L)	0.7(0.6–1.3)	0.8(0.6–0.9)	0.5(0.4–0.8)	0.22
IgG1 (mg/L)	9530.0(7530.0-11500.0)	9905.0(88102.5-12675.0)	10465.0(7480.0-12775.0)	0.71
IgG2 (mg/L)	5590.0(55000.0-66295.0)	5430.0(3720.0-6910.0)	6620.0(5397.5-9327.5)	0.37
IgG3 (mg/L)	4458.0(327.0-768.0)	717.5(4402.0-1277.5)	746.5(525.8-850.3)	0.26
IgG4 (mg/L)	5590.0(3630.0-20250.0)	9255.0(33502.5-36075.0)	12745.0(4387.5-32375.0)	0.81
T-IgE (KU/L)	311.7(162.7-787.5)	389.0(123.0-6604.0)	150.9(37.6-413.5_	0.49

### Imaging features of IgG4-RD associated heart involvement

#### Vessel distribution of IgG4- related coronary periarteritis

**Fig. 1:**
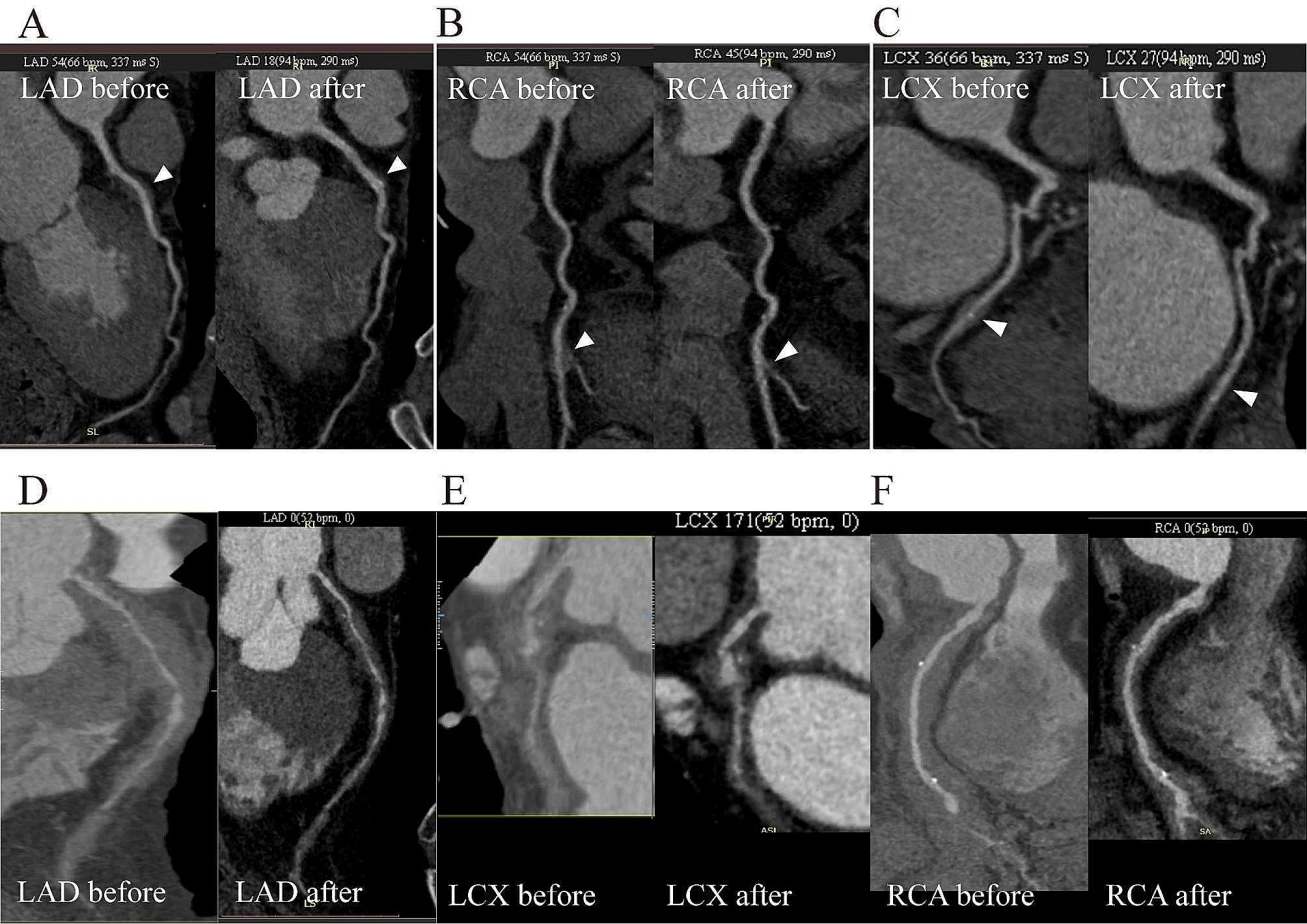
**(A-C)** Curved multiplanar reformatted coronary artery CTA demonstrate focal mural thickening and lumen stenosis (triangle) of left anterior descending artery (LAD), right coronary artery (RCA) and left circumflex artery (LCx) in a 59-year-old man before and after treatment. **(D-E)** Curved multiplanar reformatted coronary artery CTA demonstrate diffuse mural thickening and lumen stenosis of LAD, LCx and RCA in a 69-year-old man before and after treatment

**Fig. 2:**
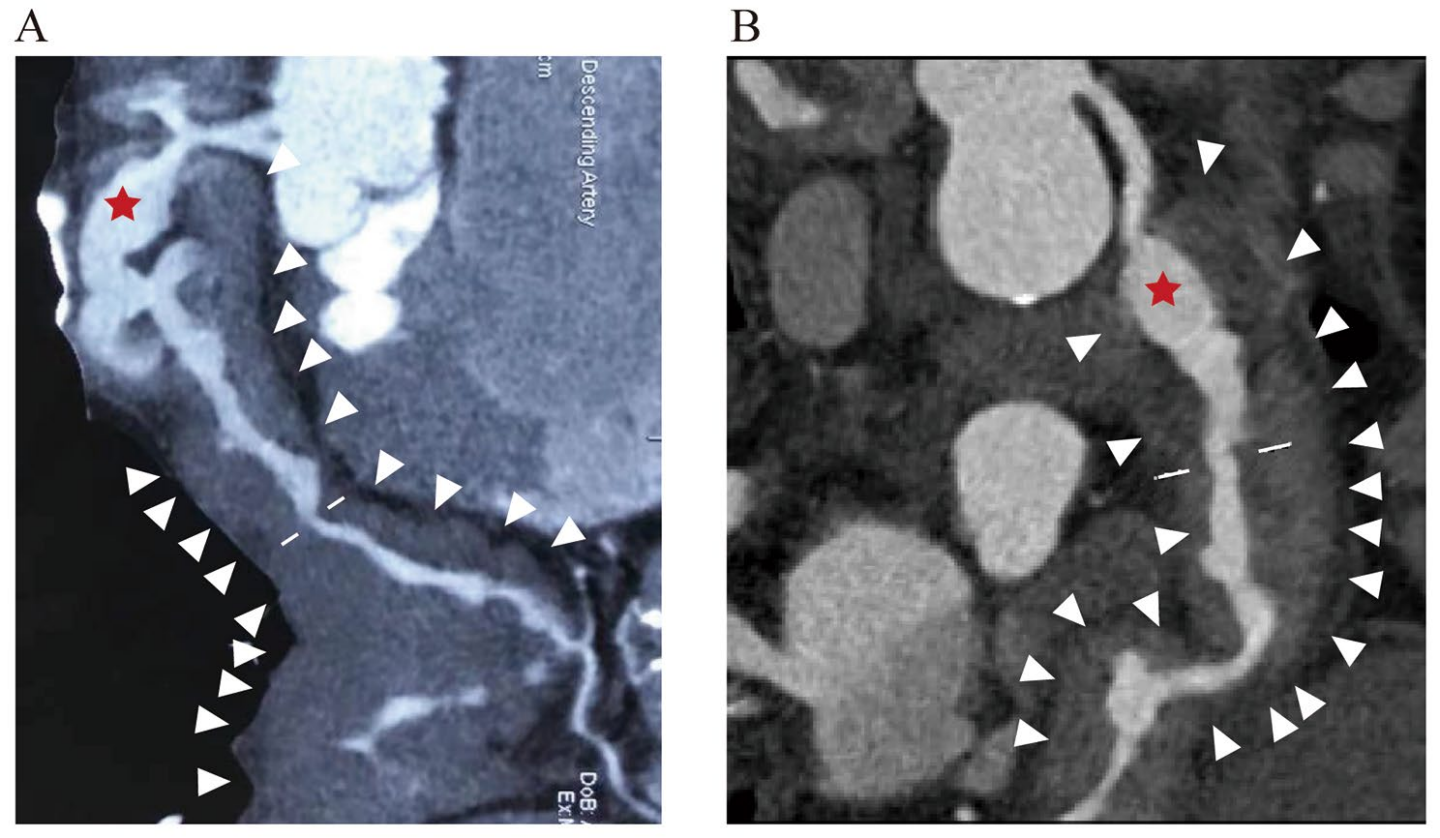
**(A)** Curved multiplanar reformatted coronary artery CTA demonstrate multifocal aneurysmal dilatation (red asterisk) and mild-to-moderate stenosis (line) of the LAD with diffuse mural thickening (triangle). Repeated contrast-enhanced CT scan with curved multiplanar reformatted **(B)** revealed radiological remission with reduction of the diffuse mural thickening of LAD after treatment with glucocorticoid

**Fig. 3:**
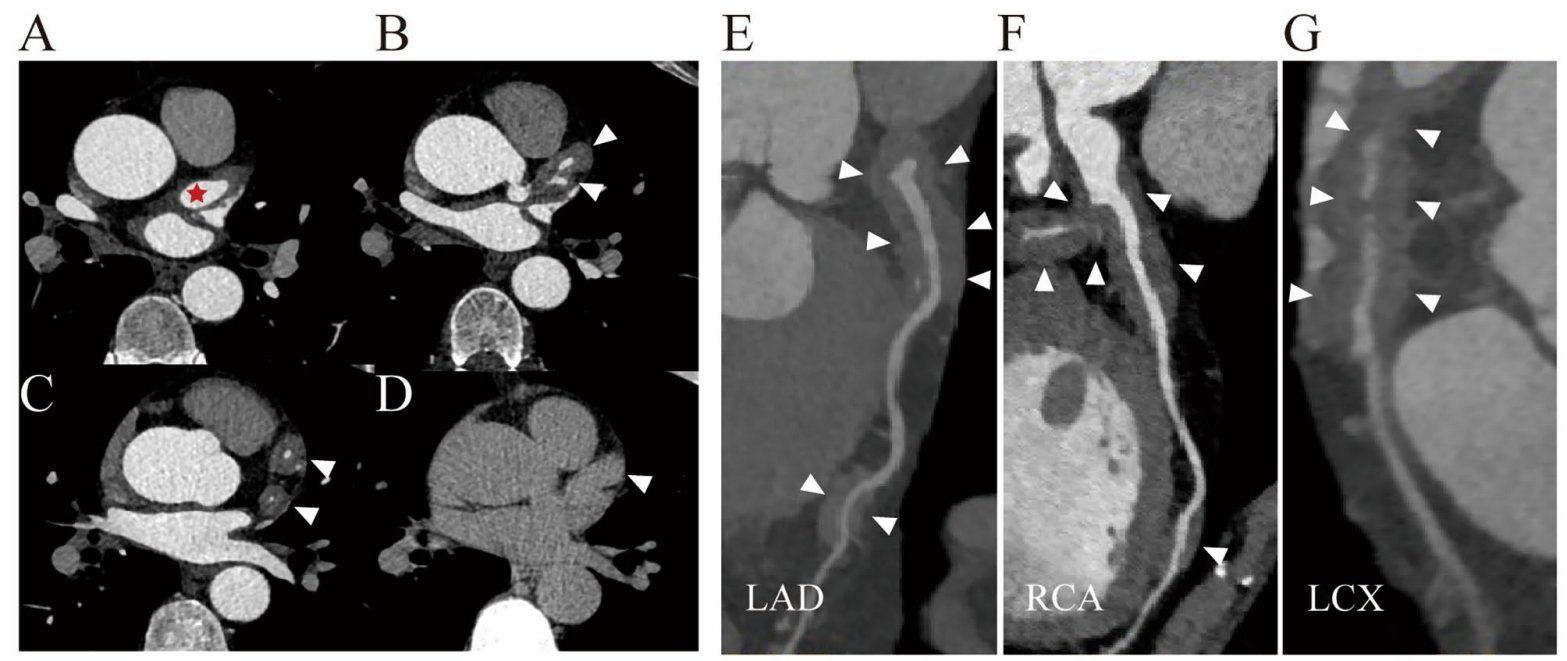
**(A)** showed aneurysmal dilatation of left main artery(LM), about 1.1 cm in diameter at its widest point (red asterisk). **(B-D)** images of the LAD and LCx showing diffuse circumferential mural thickening with associated enhancement of the wall (triangle). Curved multiplanar reformatted coronary artery CTA **(E-G)** demonstrate localized annular sheath-like soft tissue density shadow around LAD, RCA and Lcx (triangle)

Coronary periarteritis was detected by coronary CT, chest CT, PET/CT, cardiac magnetic resonance (CMR) and endovascular examinations (Figs. 1, 2 and 3). Regarding the type of coronary lesions (Tables 3 and 4), wall-thickening lesions were the most common form, observed in 12(100%) patients with coronary CTA. Coronary artery stenosis and aneurysm were also observed in 7 (58.3%) and 4 (33.3%) patients, respectively. The largest diameter of coronary aneurysm ranged from 11 mm to 21.8 mm among patients. Regarding the location of coronary artery lesions, 7 (58.3%) of the 12 patients had triple branch lesions, while only 2 patients presented with single branch lesions. Lesions in the left main coronary artery (LM), right coronary artery (RCA), left anterior descending coronary artery (LAD), and left circumflex coronary artery (LCx) were observed in 7 (58.3%), 8 (66.7%), 11 (91.7%) and 10 (83.3%) patients, respectively.

**Table 3:** Characteristics of coronary artery lesions (*N* = 12)

Coronary branch lesion counts		
Mono (%)	2	16.7%
Double (%)	3	25.0%
Triple (%)	7	58.3%
Lesion distribution		
RCA (%)	8	66.7%
LM	7	58.3%
LAD (%)	11	91.7%
LCx (%)	10	83.3%
Lesion type		
Stenosis (%)	7	58.3%
Wall thickening (%)	12	100%
Aneurysm (%)	4	33.3%

**Table 4:** Characteristics of coronary artery lesion

			LM			LAD			LCX			RCA	
SEX	Age of diagnosis	Stenosis	Wall-thickening	Aneurysm	Stenosis	Wall-thickening	Aneurysm	Stenosis	Wall-thickening	Aneurysm	Stenosis	Wall-thickening	Aneurysm
M	56		+	+	+	+		+	+		+	+	+
M	54	+	+		+	+		+	+				
M	56				+	+			+				
M	59				+	+		+	+		+	+	
M	67		+			+							
M	69				+	+	+	+	+	+	+	+	+
M	54								+			+	
F	60				+	+	+	+	+	+	+	+	+
M	47		+			+							
M	53		+			+		+	+			+	
M	55	+	+	+	+	+		+	+		+	+	
M	60		+			+			+			+	

LM, left main coronary artery; RCA, right coronary artery; LAD, left anterior descending artery; LCx, left circumfex artery

#### Imaging features of IgG4-RD associated pericarditis

**Fig. 4:**
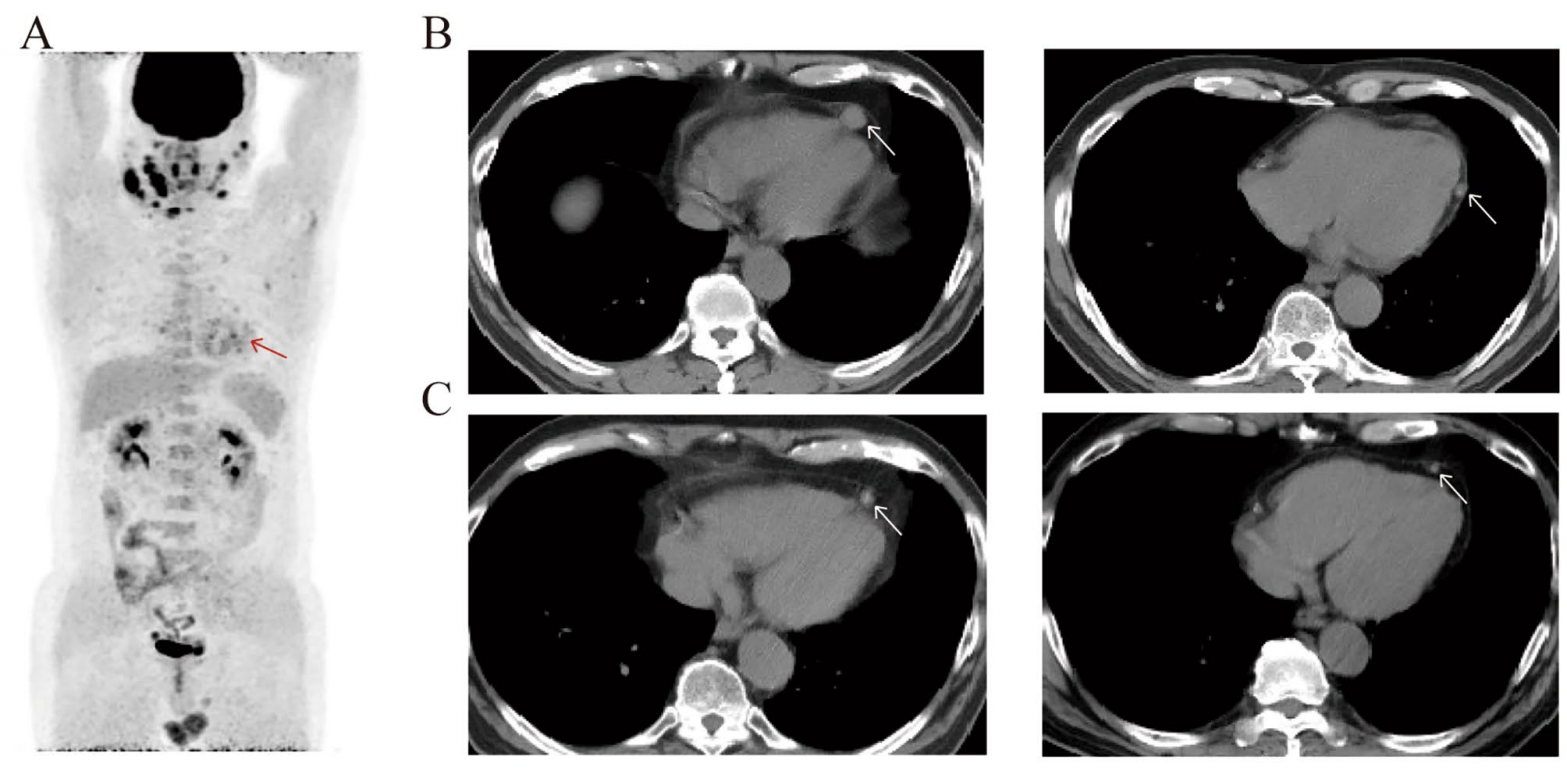
**(A)** PET/CT revealed multiple elevated radioactivity uptake in the aortic root wall, the left ventricular chamber, and the pericardial fat sac, SUVmax 5.3 (red arrow). **(B-C)** In another patient, chest CT showed multiple nodules (white arrow) of the pericardium which markedly downsized after 1 year of treatment

**Fig. 5:**
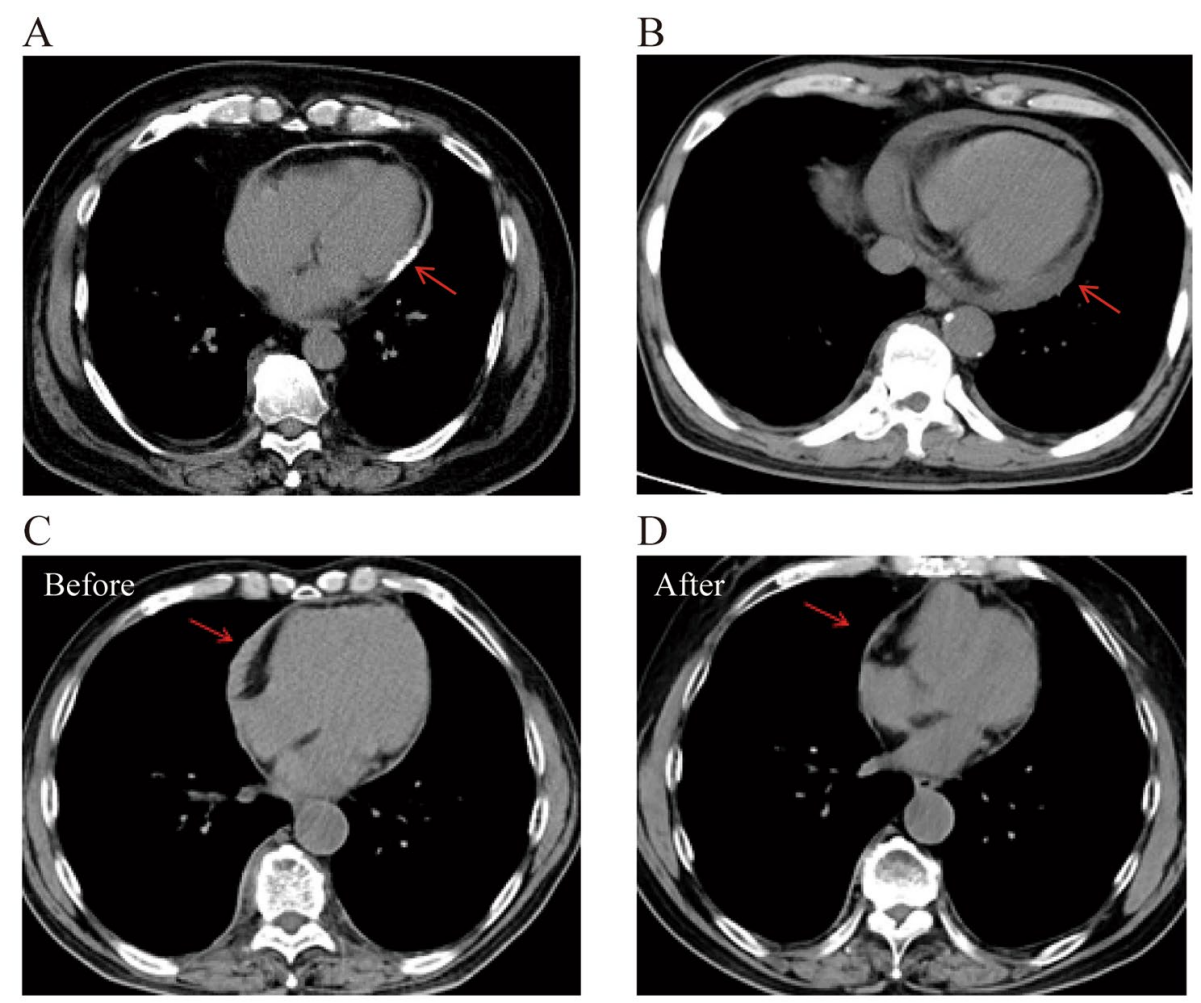
**(A)** Chest CT revealed pericardial thickening with calcification (arrow). **(B)** Chest CT indicated pericardial effusion. **(C-D)** Localized pericardial thickening (arrow) was detected and decreased

Four types of characteristic imaging features of IgG4-related pericarditis were revealed (Figs. 4 and 5), including a small amount of pericardial effusion (10 of 29, 34.5%), thickening of the pericardium (16 of 29, 55.2%), pericardium nodules (3 of 29, 10.3%) and constrictive pericarditis (1 of 29, 3.4%). Chest CT was reliable in detecting every type of pericardial involvement, while PET/CT scan showed higher standardized uptake value (SUV) of the pericardial nodules. The elevated SUV of pericardial nodules gradually dropped to normal after treatment.

#### Cardiac valves involvement

Significant valve morphology and functional abnormalities were found in 2 patients. One was a 54-year-old female diagnosed as possible IgG4-RD according to 2020 revised comprehensive diagnostic criteria and scored 34 with 2019 ACR/EULAR classification criteria, with pulmonary involvement and mediastinal lymphadenopathy. The patient showed no cardiac symptoms and recalled no medical history involving the heart including rheumatic heart disease. Her echocardiography indicated mitral stenosis with moderate to severe regurgitation. Thickened anterior and posterior leaflets of the mitral valve were observed, and adhesions were visible in the valve margin joint with decreased valve mobility. The valve orifice area was mild to moderately reduced, and the mitral valve chordae was thickened. The other patient was diagnosed as probable IgG4-RD scored 25 with ACR/EULAR criteria, with the lacrimal gland, ocular muscles, submandibular glands, sublingual lands, pleura and lymph nodes involvement. His echocardiography indicated thickened aortic valve with mild insufficiency and mitral stenosis with moderate to severe regurgitation. A new-onset mitral valve vegetation was detected during GCs plus IMs therapy and remained stable afterwards. Since there is no other explainable cause for their valve lesions, both patients were considered to have suspected IgG4-RD-associated valvular disease.

### Treatment efficacy in patients with cardiac involvement

Patients with cardiac involvement were mostly treated with glucocorticoids (GCs), GCs combined with immunosuppressant agents (IMs) or biological agents. Three (7.1%) patients received no treatment, 7 (16.7%) received GCs monotherapy, 2 received IMs monotherapy (4.8%), 26 (61.9%) received GCs plus IMs, including cyclophosphamide, mycophenolate mofetil, methotrexate and tripterygium glycosides, and 4 were treated with GCs plus rituximab (9.5%). All of the 42 patients were followed-up for more than 3 months, 100% of patients achieved reduction of IgG4-RD RI. All the patients with coronary periarteritis responded to initial treatment with symptom improvement and reduction of peri-vascular soft tissue and stenosis according to chest CT or CTA. With smaller peri-vascular soft tissue and thickening of the artery wall, luminal stenosis and even occlusion improved after treatment. Three patients relapsed. One patient had relapse at 14 months after GCs withdraw, coronary CTA showed enlarged extent of coronary artery wall-thickening. She was then given rituximab, and coronary CTA at 25 months showed disappearance of coronary artery wall-thickening and no change in stenosis. One patient had heart failure with reduced left ventricular ejection fraction (LVEF) due to ischemic cardiomyopathy. His LVEF significantly improved from 45 to 55% after 1 month of prednisone and rituximab, but had clinical relapse at 30 months with the coronary CTA showing progression of coronary artery aneurysm and stenosis, at prednisone 5 mg per day and MTX 7.5 mg per week for maintenance. One patient relapsed at 23 months of prednisone 7.5 mg per day and CTX maintenance with enlarged peri-vascular soft tissue, new-onset mild stenosis of distal LAD and enlargement of LM aneurysm (diameter 1.1 cm to 1.2 cm). All of the pericardial effusion and pericardial nodules improved after treatment, and the pericardial constriction was stable during treatment. As for the suspected IgG4-RD associated valvular disease, both patients received GCs plus IMs therapy, one had a new-onset mitral valve vegetation and no significant difference in valve abnormality was found during follow-up of the other patient.

## Discussion

We describe IgG4-RD coronary artery and pericardium involvement patterns in 42 IgG4-RD patients fulfilling ACR/EULAR classification criteria or the 2020 comprehensive diagnostic criteria for IgG4-RD. To our knowledge, this is the largest series reported to date. According to literature, cardiovascular involvements of IgG4-RD can manifest various types, including aortitis, periaortitis, periarteritis [5], pericardial [7, 8] and myocardial involvement. In our cohort, 9 patients were diagnosed with coronary periarteritis, 29 patients had pericarditis, and 4 with both coronary periarteritis and pericarditis while no myocardial and intracardiac mass lesion was addressed. 2 patients with cardiac valves involvement showed no significant improvement after IgG4-RD treatment, and no valve biopsy was obtained. Since no cardiac related medical history was recalled, we hold the opinion that these valve lesions were possibly IgG4-RD related.

Similar to other IgG4-RD patients, patients with cardiac involvements were predominantly middle-aged, male predominant, and with multiple organ lesions. 95.2% of the patients had more than one organ involved. Coronary periarteritis were more frequently accompanied with abdominal aortitis and lymph node involvement than pericarditis. Common symptoms of IgG4-related periarteritis are angina, palpitation, shortness of breath or lower extremity edema, due to myocardium ischemia or heart failure. Patients with coronary periarteritis presented with more cardiac symptoms than those with pericarditis only. Among all patients with cardiac involvement, 69% showed no symptom. Wall thickening, stenosis and aneurysm were the most common characteristic imaging findings of IgG4-coronary periarteritis. While pericardium effusion, thickening and nodules represented IgG4-pericarditis. One severe form of pericardium involvement was constrictive pericarditis, which responded poor to GCs treatment. Overall, patients with coronary periarteritis responded well to GCs. Pericardium effusion and localized pericardium thickening responded slowly to GCs and immunosuppressor treatment. Notably, no patients with pericardium effusion or thickening progressed to constrictive pericarditis in our cohort.

In a review published in 2019 that included 27 articles that described 27 patients with IgG4-related coronary peri-arteritis, the mean age was 66 years and 81% were men. The mean serum IgG4 and IgE levels were 1238 mg/dL and 2634 IU/mL, respectively. Serum CRP levels were also elevated (mean 2.95 mg/dL [normal value < 0.3 mg/ dL]). Coronary artery stenosis was identified in 67%, aneurysms in 42%, and diffuse wall thickening in 92% of patients. Notably, 22% of patients showed all 3 types of lesions. Most patients showed multiple coronary lesions, and concomitant pericardial lesions were observed in 2 patients. 33.3% coronary artery stenosis was accompanied with abdominal aortitis, higher than the prevalence of abdominal aortitis of 15.2% as reported in prospective cohorts of IgG4-RD, suggesting that coronary artery involvement may be classified as fibrotic IgG4-RD [18]. Although GCs treatment was effective, this disease can be life-threatening secondary to myocardial infarction, aortic dissection, and aneurysmal rupture. In our cohort, 100% of patients with coronary periarteritis had diffuse wall thickening, and 58.3% had triple branches lesions, involving left main coronary artery. 30.8% of patients showed concomitant pericardial lesions. All of the patients had remission under initial treatment of GCs with or without IMs. Severe cardiac ischemia and ischemic cardiomyopathy improved after immunosuppression treatment. During long-term follow-up, 3 patients with coronary involvement experienced relapse after GCs withdraw during immunosuppressor or rituximab maintenance. No aneurysmal rupture was recorded in our cohort. We believe IgG4-RD patients with cardiac symptoms should undergo echocardiograph and coronary CTA to evaluate coronary artery lesions. Also, coronary CTA should be repeated regularly during follow-up to assist therapy adjustment. Previous study have showed that the concomitant frequency of IgG4-relataed coronary peri-arteritis was substantially higher in the group with pericardial thickening than in the group without it [13]. We had a concomitant frequency of 13.8% of coronary peri-arteritis involvement in patients with pericardial involvement as well. This suggests that pericardial thickening might reflects the intense inflammation of the coronary artery. And patients with IgG4-RD complicated with pericardial thickening should undergo a more extensive workup, including echocardiograph and coronary CTA.

Pericardial thickening with effusion or constrictive pericarditis [19] was reported as a rare manifestation of IgG4-RD which could be accompanied by mediastinal infiltrative soft tissue lesion and pleural effusion [20]. In one recent review of IgG4-RD with pericardial involvement, 32 published cases were included [21]. The mean age was 64 years and 65.7% of patients were males. IgG4-related pericarditis was mostly associated with pleural involvement. In most reported cases, a pericardial biopsy was done to support the diagnosis of IgG4-RD. Pericardial fluid analysis could be predominant lymphocytes [22, 23]. Pericardial pathology was reported similar to other IgG4-RD organ pathology with marked storiform fibrosis with excess lymphoplasmacytic infiltrate, while immune-histochemistry showed an increased number of IgG4-possitive plasma cells. In our cohort, the demographic characteristics are the same as previously reported cases. Also, all the patients had elevated serum IgG4. Pericardial effusion and thickening responded well to GCs and IMs treatment. Most of the patients in our center were asymptomatic with mild effusion or pericardium thickening which didn’t lead to the decision of performing pericardiotomy. This was also the reason that no pericardial biopsy was referred to as diagnostic evidence. In the case of pericardial involvement, obtaining a biopsy can be challenging and requires an invasive procedure. We agree to the previous perspective that the decision to perform a pericardial biopsy should be considered on a case-to-case basis, maybe restricted to cases with a severe clinical presentation that requires surgical intervention. As suggested in the previous literature, along with medical treatment, pericardiotomy is a possible relieving procedure that proved to be effective in IgG4-RD with pericardial involvement, especially in patients with constrictive pericarditis.

According to previous study, valvulitis can be rare clinical manifestation of IgG4-RD and can precede other symptoms [12]. Severe calcific aortic and mitral valve disease was reported, following by rapid failure of bioprosthetic replacement requiring repeated intervention. IgG4-positive plasma cell filtration has been described in mitral valve of IgG4-RD patient, and aortic valve involvement has been observed in a few patients with other systemic manifestations of the disease [24, 25]. Although only 2 patients with suspicious valvulitis related to IgG4-RD were included, we believed that with proper screening by echocardiography in patients with cardiac symptoms might discover more cases and lead to treatment in time.

Our study had several limitations. This was a bi-centric study with a relatively small population. To avoid selection bias, we estimated the prevalence based on data from the general hospital only. Pathological confirmation of IgG4-RD was obtained from extra-cardiac organ biopsies, few underwent cardiac biopsy because of the challenge in invasive procedure. Due to the lack of medical resources and patients concerns about enhanced CT, not all patients had repeated coronary CTA during follow-up. Furthermore, the diagnostic criteria for cardiac involvement included treatment response to better address IgG4-related cardiac involvement. No patients with coronary arteritis were excluded because of lack of treatment response. For pericardial effusion or thickness, 64 patients were excluded because no treatment response was observed. We had to do so for that pericardial biopsy was difficult to obtain. We could have overlooked patient with poor treatment response.

In conclusion, heart involvements in IgG4-RD are rare, with diverse clinical presentations and sometimes life-threatening. Delayed diagnosis and treatment may lead to irreversible consequences. Cardiac involvement in IgG4-RD is easily overlooked since many patients are asymptomatic, screening for cardiovascular involvement in IgG4-RD patients should be emphasized. Patients showed a satisfactory response to glucocorticoid based treatment, with relieved symptoms, and rehabilitated radiological findings.

### Electronic supplementary material

Below is the link to the electronic supplementary material.

Supplementary Material 1

## Data Availability

The data that support the findings of this study are not openly available due to reasons of sensitivity and are available from the corresponding author upon reasonable request. Data requests can be made to Wen Zhang via this email: zhangwen91@sina.com. Data are located in controlled access data storage at Peking Union Medical College Hospital.
