# Supplementary material for: Coronary periarteritis and pericarditis are rare but distinct manifestations of heart involvement in IgG4-related disease: a retrospective cohort study
Source: Orphanet J Rare Dis. 2024 Jul 15;19:266. doi: 10.1186/s13023-024-03266-y (PMC11247878; doi:10.1186/s13023-024-03266-y)
Supplement: Supplementary file 1 — Supplementary Material 1 [file 13023_2024_3266_MOESM1_ESM.docx]

Supplementary Table 1 Demographic parameters and organs involvement of IgG4-RD

patients with/without Cardiac involvement

|  | Cardiac（n = 42) | Non-Cardiac (n = 1033) |
| --- | --- | --- |
| Male ( % ) | 78.6% | 61.9% |
| Age of diagnosis,years | 58.2±12.8 | 52.9±13.9 |
| Involved organs | 3（2-4.5） | 3 (2 - 4) |
| Allergy history | 18/42（43.9%） | 519/1033(51.9%) |
| Other organs affected |  |  |
| Pancreas | 11/42（26.1%） | 385/1033(37.3%) |
| Submandibular glands | 23/42（54.7%） | 605/1033(58.6%) |
| lacrimal glands | 14/42（33.3%） | 498/1033(48.2%) |
| Cholangitis | 6/42（14.3%） | 199/1033(19.3%) |
| Sinus | 6/42（14.3%） | 278/1033(26.9%) |
| Abdominal aortits | 8/42（19.0%） | 106/1033(10.3%) |
| Lung | 11/42（26.2%） | 249/1033(24.1%) |
| Lymph nodes | 18/42（42.9%） | 452/1033(43.8%) |
| EULAR ＞20 | 30/42(71.4%) | 690/1033(66.8%) |
